# Diffusion tensor imaging based multiparametric characterization of renal lesions in infants with urinary tract infections: an explorative study

**DOI:** 10.1186/s12887-021-02769-y

**Published:** 2021-10-08

**Authors:** Yvonne Simrén, Eira Stokland, Sverker Hansson, Hanna Hebelka, Par-Arne Svensson, Kerstin M. Lagerstrand

**Affiliations:** 1 Department of Radiology, Institute of Clinical Sciences at Sahlgrenska Academy, University of Gothenburg, Gothenburg, Sweden; 2 Department of Pediatrics, Institute of Clinical Sciences at Sahlgrenska Academy, University of Gothenburg, Gothenburg, Sweden; 3 Department of Radiation Physics, Institute of Clinical Sciences at Sahlgrenska Academy, University of Gothenburg, Gothenburg, Sweden

**Keywords:** Diffusion tensor imaging, Infant, Kidney, Pyelonephritis, Urinary tract infection

## Abstract

**Background:**

Conventional diffusion weighted imaging (DWI) is a promising non-invasive tool in the evaluation of infants with symptomatic urinary tract infections (UTI). The use of multiparametric diffusion tensor imaging (DTI) provides further information on renal pathology by reflecting renal microstructure. However, its potential to characterize and distinguish between renal lesions, such as acute pyelonephritic lesions, permanent renal damages or dysplastic changes has not been shown. This study aimed to evaluate the potential of multiparametric DTI for characterization of renal lesions with purpose to distinguish acute pyelonephritis from other renal lesions in young infants with their first UTI.

**Methods:**

Nine kidneys in seven infants, age 1.0–5.6 months, with renal lesions i.e. uptake reductions, on acute scintigraphy performed after their first UTI, were included. The DTI examinations were performed during free breathing without sedation. The signal in the lesions and in normal renal tissue was measured in the following images: b0, b700, apparent diffusion coefficient (ADC), and fractional anisotropy (FA). In addition, DTI tractographies were produced for visibility.

**Results:**

There was a difference between lesions and normal tissue in b700 signal (197 ± 52 and 164 ± 53, *p* = 0.011), ADC (1.22 ± 0.11 and 1.45 ± 0.15 mm^2^/s, *p* = 0.008), and FA (0.18 ± 0.03 and 0.30 ± 0.10, *p* = 0.008) for all nine kidneys. Six kidneys had focal lesions with increased b700 signal, decreased ADC and FA indicating acute inflammation. In three patients, the multiparametric characteristics of the lesions were diverging.

**Conclusion:**

Multiparametric DTI has the potential to further characterize and distinguish acute pyelonephritis from other renal lesions in infants with symptomatic UTI.

## Background

Urinary tract infection (UTI) is a common disease in infants with an incidence of 2% in children under 1 year of age [1]. Ultrasound is often used as a first line investigation of infants with UTI. The use of further imaging varies depending on which protocol that is used. When further imaging is needed, for example to evaluate the extent of engagement of the renal tissue or to identify complications of the infection, dependent on availability, 99mTc-Dimercaptosuccinic acid (DMSA) scintigraphy, computed tomography (CT) or contrast enhanced magnetic resonance imaging (MRI) can be used. However, DMSA scintigraphy and contrast-enhanced techniques are invasive as they require an intravenous injection. DMSA scintigraphy and CT also has the drawback of using ionizing radiation. Therefore, diffusion weighted imaging (DWI), a non-invasive MRI-technique, has been suggested as an alternative method in the evaluation of children with symptomatic UTI [2–6].

Renal parenchymal lesions detected in imaging of younger infants with an UTI may have different etiologies, such as acute inflammation i.e. acute pyelonephritis, permanent renal damage or dysplastic changes that are important to differentiate as it will impact the clinical management of the patient. DWI in multiple direction, i.e. diffusion tensor imaging (DTI), can give an insight into the microstructure of the renal tissue as the use of multiple diffusion directions better depicts the highly radiating oriented renal structure [7–12]. This technique enables calculation of several quantitative measures that can be used in the diagnosis together with the findings from the visual evaluation. The comprehensive analysis of multiple DTI parameters i.e. multiparametric DTI, can provide further information that may improve the characterization of renal lesions in infants with symptomatic UTI. Recent studies have shown the potential of the technique to evaluate renal pathology in older children [13–17]. It has also been shown that free breathing DTI is feasible and can generate images with higher quality compared to conventional DWI in children with acute pyelonephritis [18]. To our knowledge, there are no studies on renal DTI in young infants, a patient group that would benefit from the use of this non-invasive, non-radiating, short scan technique. The potential of DWI for detection of acute pyelonephritis has recently been shown in non-sedated free breathing infants with symptomatic UTI [6]. However, in several infants’ lesions the findings were equivocal and not typical for acute pyelonephritis raising the need for further characterization of such lesions.

**Aim:** To study the potential of multiparametric DTI for characterization of renal lesions with purpose to distinguish different renal lesions in young infants with their first UTI.

## Methods

### Patients

This explorative study was performed in conjunction with a recently published study [6] including infants (< 6 months of age) that had been examined with acute DMSA scintigraphy as part of the clinical routine follow-up of infants with first time community-acquired symptomatic UTI in our hospital over a period of 19 months. The MRI examinations were performed within two weeks of the acute DMSA scintigraphy. Fourteen patients with uptake reductions on the DMSA images were eligible for the study. Seven patients were included as they had DWI as well as DMSA images of high diagnostic quality without artefacts.

Written informed consent for participation in this study was obtained from the guardians of the children. The study was approved by the Swedish Ethical Review Authority in Gothenburg, Gothenburg, Sweden (Dnr 214–11).

### Imaging methods

The MRI examination was performed in median 1 day (range 0–12 days) from onset of the infection and 1 day from the acute DMSA examination (range 0–7 days). All infants underwent MRI examinations, sleeping or resting in the scanner after feeding, without any use of sedation or anesthetics. The scanning was performed during free breathing using a 1.5 T MRI system (GE Signa HDx, Twinspeed, GE Medical systems, Waukesha, WI, USA) with a 40 mT/m maximum gradient capability using the body coil as radiofrequency transmitter and the eight-channel cardiac coil as radiofrequency receiver. The total examination time was 15 min, with an effective acquisition time of under 5 min, including a prescan and a T2 weighted scan for verification of morphological findings and volume measurements. The acquisition time of the DTI scan was 58 s.

The DTI scanning was performed using coronal and fat saturated single-shot spin-echo planar 2D imaging, TR = 2000 ms, TE = 66 ms, acceleration factor = 2, b-value = 0 and 700 s/mm^2^, NEX = 1, 24 diffusion directions. Ten coronal slices with a thickness of 6 mm and a scan pixel size of 2.2 × 1.8 mm^2^ were used to cover both kidneys. For all infants, saturation bands were used to suppress regions with moving structures and thereby reduce the appearance of artifacts in the DWI scans. For calculation of b700, all diffusion directions from the b700 s/mm^2^ acquisitions were used. Apparent diffusion coefficient (ADC) maps were calculated using a monoexponential model. DTI, ADC, fractional anisotropy (FA) maps and tractographies were reconstructed on the workstation of the MRI system (Advantage Workstation, GE Healthcare, USA). The tractographies were launched from seeds within a ROI including the whole kidney on the coronal slice with findings on the b700 image. The FA threshold (0.05–0.1) and the maximum angle threshold (55°–100°) for the tractography were chosen to get high density of tracks with limited spurious tracks outside the boundaries of the kidneys [13].

The DMSA examinations were performed in accordance with the guidelines of the Pediatric Committee of the European Association of Nuclear Medicine [19]. All DMSA images were reviewed by a senior nuclear medicine specialist. The criterion for lesions on the DMSA images was the presence of uptake reductions. Follow-up DMSA examinations were performed 1 year after the first DMSA examination as part of the clinical routine.

### Image analyses

The descriptive image evaluation was performed on a diagnostic workstation with adjustment of the optimal window setting on a case-by-case basis. Two senior pediatric radiologists reviewed the images in consensus. The image sets were evaluated with regard to signal changes in the renal parenchyma corresponding to the lesions detected on the acute DMSA images. The images were also evaluated for any morphological findings such as hydronephrosis or duplications. The pattern of the tracks from the tractography was evaluated for any presence of distortion or reduction of tracks.

Quantitative image analyses were performed by a senior pediatric radiologist using Image J (*1.45 s*, National Institute of Health, USA). Renal volume estimation was performed after manually outlining the renal contour on all sliced on the T2 weighted images. For estimation of quantitative parameters, circular regions of interest (ROI), with an area of 50 mm^2^, were manually placed on the DTI images, as well as on the ADC and FA maps. All ROIs were positioned to avoid large vessels, the renal calyces, or renal pelvis. The lesions were evaluated with three ROIs placed inside the centre of the lesions using the acute DMSA images as a reference to verify the location. For the evaluation of normal renal tissue in unilateral lesions, ROIs were placed in both the apical, lateral and the caudal part of the contralateral kidney in. In patients with bilateral lesions, three ROIs were instead placed in areas of the same kidney where there were no uptake reductions on the DMSA scans or signal changes on the DTI images. The mean value of the three ROIs were used for further analyses. The multiparametric analysis was performed by inspecting the combined data from the descriptive and quantitative analyses, with the intention to find patterns among the characteristics of the different lesions.

### Statistical analyses

The statistical analyses were performed in IBM SPSS Statistics 26 software package. For comparison of values between lesions and normal renal tissue, the Wilcoxon signed rank test was used and *p*-values of < 0.05 were considered significant.

## Results

### Patients

DTI images from a total of seven infants, median age 1.2 months (range 1.0–5.6 months), were analysed. Two of the patients had bilateral lesions, resulting in a total of nine kidneys evaluated. Table 1 shows patient characteristics for the seven patients.

**Table 1:** Patient characteristics for seven patients with renal lesions on the acute DMSA images

Patient no	Sex	Age (months)	Bacteriuria	Max temp (°C)	Max CRP (mg/L)	Laterality of renal lesion	Vesico-ureteral reflux grade Right/Left	Renal volume (ml) Right/Left	Follow-up DMSA
1	Boy	> 1.2	*E. coli*	39.1	91	Left	VCUG not performed	28/35	Normal
2	Girl	≤1.2	*E. coli*	38.7	44	Left	0/4	18/17	Persistent lesions
3	Boy	> 1.2	*E. coli*	37.2	8	Left	0/0	14/14	Normal
4	Girl	≤1.2	*E. coli*	38.3	62	Bilateral	0/0	23/22	Persistent lesions
5	Boy	≤1.2	*E. coli*	38.6	147	Bilateral	0/0	29/26	Normal
6	Boy	> 1.2	*E. coli*	40.3	90	Left	0/0	18/20	Persistent lesions
7	Girl	≤1.2	*Enterococci*	38.3	180	Right	2/0	17/19	Persistent lesions

*VCUG* voiding cystourethrography, *DMSA* 99mTc-Dimercaptosuccinic acid scintigraphy

### Descriptive characteristics

**Fig. 1:**
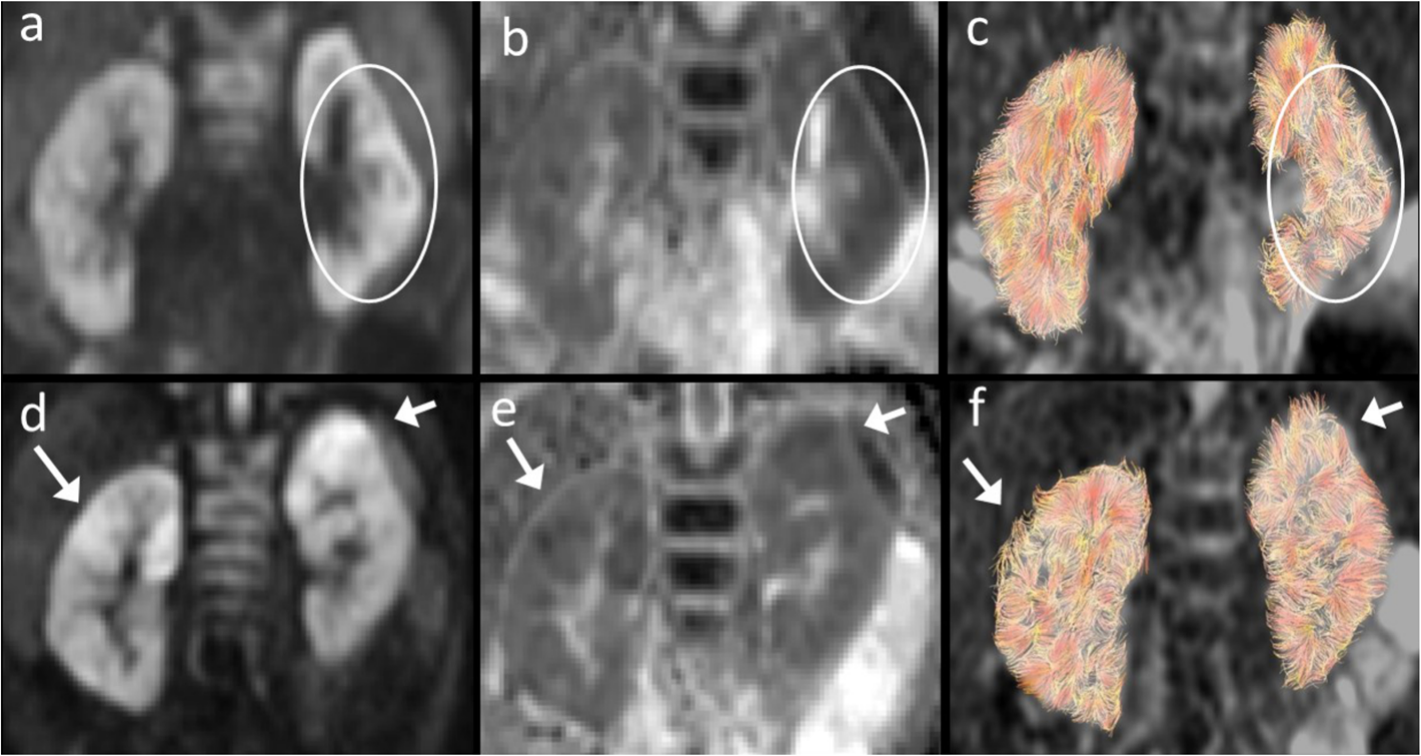
Example of two infants, no 2 **a**–**c** and 4 **d**–**f**, with lesions (circles/arrows) showing the characteristics of increased signal on the b700 **a**, **d**, reduced ADC **b**, **e** and distorted/less tracks on tractographies **c**, **f**

Table 2 shows the descriptive characteristics of the findings on the images for the different methods. The morphological evaluation revealed one patient (no 7) with thinner parenchyma in the apical part of the kidney, but no hydronephrosis, duplex or other malformations were found in any of the other kidneys. Focal increased signal on b700, corresponding to decreased ADC and distorted/less tracks on tractography, was seen in patient no 1, 2, 4 and 5. To exemplify the findings of this group, the images of patients no 2 and 4 are shown in Fig. 1.

**Table 2:** Descriptive characteristics of the findings on the images b0, b700, ADC and Tractography compared to normal tissue

Patient no	Laterality	DMSA uptake reduction	b0 signal	b700 signal	ADC value	Tractography tracks
1	Left	Apical	Apical +	Apical +	Apical −	Distorted/Reduced
2	Left	General	No change	General +	General −	Distorted/Reduced
3	Left	Apical	No change	General +	General −	Patchy distorted pattern/Reduced
4	Left	Apical	Apical +	Apical +	Apical −	Distorted/Reduced
4	Right	Apical	No change	Apical +	Apical −	Distorted/Reduced
5	Left	General	No change	Apical +	Apical −	Distorted/Reduced
5	Right	Apical	No change	Apical +	Apical −	Distorted/Reduced
6	Left	Apical	Apical −	Apical mix +/**−**	Apical −	Distorted/Reduced
7	Right	General	Apical −	No change	Apical −	Distorted/Reduced

*DMSA* 99mTc-dimercaptosuccinic acid scintigraphy, *ADC* apparent diffusion coefficient

+ increase, **−** decrease

**Fig. 2:**
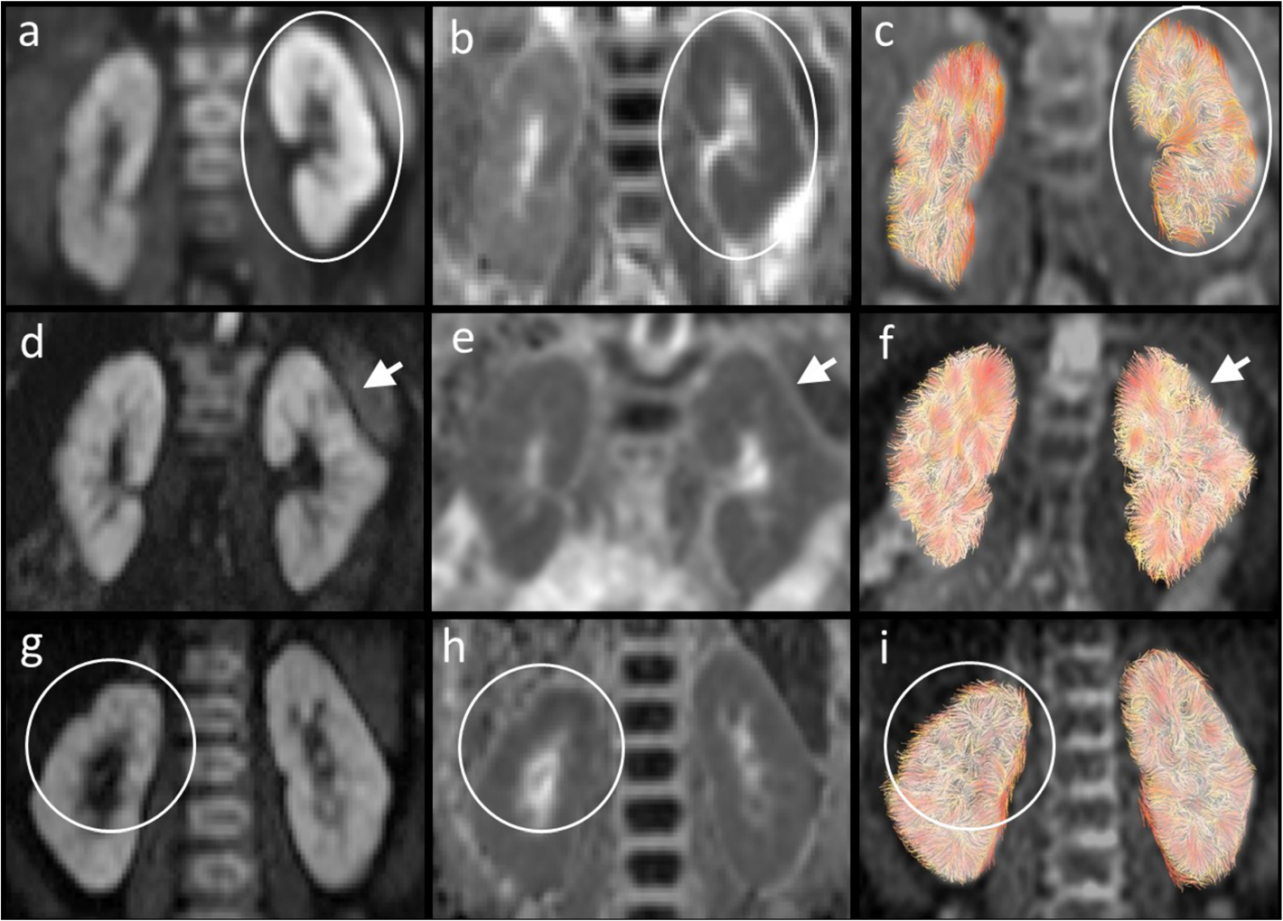
Illustration of the patients (no 3, 6 and 7) with diverse DTI characteristics on the diffusion dependent images b700 **a**, **d**, **g**, ADC **b**, **e**, **h** and tractography **c**, **f**, **i**. In patient no 3 (**a–c**) generalized changes were seen in the left kidney with increased b700, decreased ADC and patchy changes of the tracks on the tractography. In patient no 6 **d–f** there was an apico-lateral discrete increased signal with a central small low signal area (arrow) on the b700 of unclear etiologic. Distorted track pattern could be seen on the tractography in this area. In patient no 7 **g**–**i** there was a decreased ADC signal in the upper half of the right kidney showing less tracks with some distortion, but no signal increase could be depicted visually on the b700

In patients no 3, 6 and 7 (Fig. 2), the patterns were more diverse and differed regarding either the signal intensity on the different images or distribution of the findings.

## Quantitative characteristics

Table 3 shows quantitative characteristics expressed as the difference in values between lesions and normal tissue. For patient no 6, the values were obtained from ROIs placed in the lesion but avoiding the small central area with reduced signal. There was a significant increase in signal between lesions and normal tissue for b700 (197 ± 52 and 164 ± 53, *p* = 0.011) and corresponding decrease for ADC (1.22 ± 0.11 and 1.45 ± 0.15 mm^2^/s, *p* = 0.008) and FA (0.18 ± 0.03 and 0.30 ± 0.10, *p* = 0.008). No significant difference in the b0 signal between lesions and normal tissue was found (460 ± 91 and 458 ± 153, *p* = 0.375)

**Table 3:** Difference in b0, b700, ADC and FA in lesions relative normal renal tissue in nine kidneys in seven patients

Patient no	Laterality	b0 signal diff	b700 signal diff	ADC diff	FA diff
1	Left	21%	32%	−12%	− 44%
2	Left	−2%	26%	−19%	−35%
3	Left	3%	38%	− 25%	− 56%
4	Left	21%	33%	−22%	−35%
4	Right	11%	24%	−20%	− 38%
5	Left	0%	22%	−11%	− 34%
5	Right	8%	25%	−14%	−38%
6	Left	−2%	19%	−7%	− 36%
7	Right	− 23%	−3%	−13%	−35%

*ADC* apparent diffusion coefficient, *FA* fractional anisotropy, *diff* difference between lesion and normal tissue

## Multiparametric analyses

All patients showed decreased FA and ADC value in lesions compared to normal tissue. In all patients, the decrease in FA was larger than the decrease in ADC. Kidney no 1, 2, 4 left, 4 right, 5 left, 5 right showed the pattern of focal lesions with distorted/less tracks on the descriptive analyses with corresponding quantitative findings of increased b700 signal, decreased ADC and FA.

Three patients differed from this pattern. Patient no 3 showed more generalized changes on the descriptive analysis and a larger difference between lesion and normal tissue for all three quantitative parameters compared to the other patients. In patient no 6, the discrete signal changes that surrounded the small focal lesion followed the pattern of patient no 1, 2, 4 and 5 but the small focal lesion had low signal on b700. In contrast to all other patients, no 7 showed a decrease in ADC and FA but no corresponding increase in the b700 signal compared to normal tissue.

## Discussion

This explorative study investigated the potential of multiparametric DTI in characterization of renal lesions in infants with their first UTI. The typical findings of acute pyelonephritis using conventional DWI has been described as areas of hindered diffusion [2–5]. The same findings have been reported using DTI [18]. Multiparametric DTI provides several parameters that can be used for further characterization of these findings. By combining these parameters, the lesion could be characterized further and lesion patterns were found. This complementary information may be valuable in clinical practise as it might be used to distinguish acute pyelonephritis or abscess from permanent renal damage or dysplastic changes.

In six of the nine kidneys, we found a pattern of focal hyperintense lesions on b700 with decreased ADC indicating hindered diffusion in the affected tissue. The lesions also showed alterations in anisotropy, confirmed by significant changes in FA. We think it is likely that this pattern is typical for acute pyelonephritis. There is limited knowledge on the pathophysiological mechanisms accounting for the abnormalities found in imaging of pyelonephritic changes but it is suggested to be multifactorial, with the inflammatory response causing oedema and accumulation of intravascular and intratubular granulocytes with secondary focal ischemia [20, 21]. We hypothesize that the findings in our study with diffusion restriction as well as the reduced FA could be explained by these inflammatory changes. The fact that the decrease in FA between the lesions and normal parenchyma was greater than the decrease in the ADC could imply that the highly radially oriented tubules in the kidney makes the FA more sensitive to cell infiltration in the tubules compared to ADC [8]. This would eventually also lead to loss of the normally radiating pattern that could be seen on the tractographies in these six kidneys. In concordance with our expectations, there was no significant difference in the b0 signal in the lesions compared to normal tissue.

In the other three patients (no 3, 6 and 7), the patterns differed from the group of patients with more typical pyelonephritic pattern. In patient no 3, more generalized changes in the parenchyma was seen and with a larger difference between lesion and normal tissue for all three quantitative parameters compared to the other patients. There was no increase in renal volume that indicated renal swelling and clinically the inflammatory parameters were low; thus, the nature of the finding is unclear. However, the etiology cannot be determined without histopathological correlation. In patient no 6, the descriptive characteristic of the lesion was discrete hyperintensity that might have been overseen visually but showed typical pattern of acute pyelonephritis when evaluated further with quantitative measures. The central low signal area on the b700 was difficult to characterize, due to its small size but the signal pattern could suggest a minimal remnant abscess or bleeding. In contrast to the other patients, no 7 showed a decrease in ADC and FA in the lesion compared to normal parenchyma, but no corresponding increase in the b700 signal. This low diffusion signal (b700) can to some extent be the result of a very low T2 signal (observed on the b0 images) as the b700 signal reflects both the diffusion and the T2 signal in the tissue. The radial pattern on the tractography was also highly disturbed. The thin parenchyma in the affected part of the kidney, together with findings that were persistent on the follow-up of the patient, suggests that these changes most probably were of hypoplastic/dysplastic type. We suggest that the low b700 signal could indicate that there was no acute edema and that this lesion was in fact permanent.

Previous studies have shown that DTI is a valuable tool to evaluate renal pathology in the pediatric population [14–18]. Reports have suggested that DTI-based parameters, including ADC and FA, are potential biomarkers in the evaluation of kidneys with ureteropelvic junction obstruction [14, 16]. DTI has also been described as a promising non-invasive method in the assessment of renal allograft health in pediatric allograft recipients [17]. There are a few reports on the potential of DTI to provide complementary information to DWI in assessment of various focal lesions in the adult and pediatric populations [11, 13, 22] but the value of using DTI in the diagnosis of acute pyelonephritis is sparsely studied. Lair et al. studied 31 children (age 6 months of age to 16 years) with suspected acute pyelonephritis with the objective to compare image quality in free breathing DTI compared to respiratory triggered conventional DWI [18]. They found that DTI demonstrated significantly better image quality and showed agreement with DWI in all patients. Although the goal of the study was not to assess the diagnostic value of DTI, they concluded that DTI could replace DWI in the diagnosis of acute pyelonephritis.

The use of free breathing methods in children is attractive since MR examinations in children is demanding due to long scan times and a need for sedation. Infants under 6 month of age can often be scanned during sleep after feeding if an optimized scan protocol is used. In this study we used a short scan protocol that was optimized for non-sedated free breathing scanning with an acquisition time for the DTI scan of under 1 min. This is a great advantage in younger infants and increases the clinical usability of the method. Moreover, the ability of MR methods to detect or rule out confounding pathologies, such as duplications or other malformations, is another strength and limits the need for further imaging.

However, the use of a scan protocol optimized for non-sedated free breathing scanning in combination with the thin cortex in young infants did not allow for adequate differentiation between the cortex and medulla for separate analyses. In addition, the small kidney size in younger infants makes the ROI placement in the lesions and normal tissue challenging. Therefore, ROIs with small areas were used to improve the accuracy of the placement. This study was designed as an explorative study with limited number of patients. Even though we found that further characterization of the lesions could indicate either acute pyelonephritis or alternative etiology, further studies are needed to understand the relationship with specific diagnoses. The significance of the observations and the use of the method in clinical practise also needs to be explored.

## Conclusion

Multiparametric DTI showed a diversity in renal lesions in young infants with UTI. With the use of multiparametric analyses, the lesions could be characterized further and lesion patterns could be found. Thus, multiparametric DTI has the potential to distinguish acute pyelonephritis from other renal lesions, such as permanent renal damages or dysplastic changes. As a non-invasive, non-radiating, short scan technique, multiparametric DTI is an attractive method that may provide valuable information for the evaluation of these patients in clinical practice.

## Data Availability

The analysed data sets generated during this study are available from the corresponding author on reasonable request.

## Abbreviations

ADC: Apparent diffusion coefficient;
CT: Computed tomography
DMSA: 99mTc-Dimercaptosuccinic acid
DTI: Diffusion tensor imaging
DWI: Diffusion weighted imaging
FA: Fractional anisotropy
MRI: Magnetic resonance imaging
UTI: Urinary tract infections
